# Bioactive Compounds in Seafood: Implications for Health and Nutrition

**DOI:** 10.1002/fsn3.70181

**Published:** 2025-04-20

**Authors:** Tabussam Tufail, Huma Bader Ul Ain, Jawad Ashraf, Sammina Mahmood, Sana Noreen, Aiman Ijaz, Ali ikram, Muhammad Tayyab Arshad, Muhammed Adem Abdullahi

**Affiliations:** ^1^ School of Food Science and Engineering Yangzhou University Yangzhou China; ^2^ School of Food & Biological Engineering Jiangsu University Zhenjiang China; ^3^ University Institute of Diet and Nutritional Sciences The University of Lahore Lahore Pakistan; ^4^ Department of Botany, Division of Science and Technology University of Education Lahore Pakistan; ^5^ University Institute of Food Science and Technology The University of Lahore Lahore Pakistan; ^6^ Department of Food Science and Postharvest Technology Jimma University College of Agriculture and Veterinary Medicine, Jimma University Jimma Ethiopia

**Keywords:** bioactive components, functional food, seafood

## Abstract

The significance of Seafood as a reservoir of bioactive substances is increasing. With sea creatures making up approximately half of all living organisms on the planet, seas, and oceans present many innovative materials and are believed to hold the most substantial remaining reserve of beneficial natural compounds. Seafood provides a plentiful supply of essential nutrients, including high‐quality protein, various fatty acids (such as omega‐3s), and bioactive compounds like taurine, carotenoids, and phytosterols, all contributing to its numerous health advantages. Furthermore, seafood contains bio‐lipopeptides, polysaccharides, and phenolic compounds, and it promotes health through its antioxidant and anti‐inflammatory effects. Enzymes, vitamins, and minerals further enrich its nutritional profile, supporting various metabolic processes and overall well‐being. This review emphasizes the health benefits of seafood consumption, encompassing its cardio‐protective effects that bolster heart health, its antidiabetic properties that aid in regulating blood sugar levels and its anti‐cancer effects that may lower the risk of specific cancers. Additionally, seafood contributes to anti‐obesity effects, enhances brain health, delivers antioxidative activity to combat oxidative stress, and supports maternal care during pregnancy and lactation.

## Graphical abstract

Seafood is vital in promoting cardiovascular health, regulating blood sugar levels, reducing the risk of specific cancers, combating obesity, enhancing brain health, and supporting maternal care during pregnancy and lactation. Given the rising interest in functional foods and nutraceuticals, I believe this review will be particularly interesting to your readers, providing a comprehensive overview of the diverse health benefits of seafood consumption.

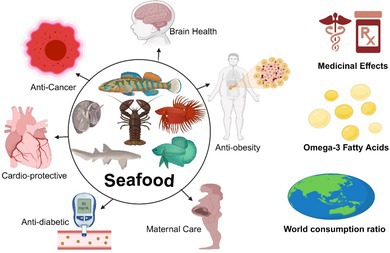

## 1 Introduction

The expression “bioactive” is made up of bio and active. In the historical background, Bio is from the Language of Greek (βίο‐) “bios” (life), which alludes to life. What is more—dynamic from Latin “actives,” implies lively, ready to go, with vitality or includes a movement. That movement presents every one of the peculiarities from a scientific sense (Bernard 2011). Diet plays a hugely helpful part in fighting human diseases. Late improvements in nutraceuticals and useful functional foods have affirmed the availability of bioactive parts in human eating routines. Fish stood out over the most recent very long while because of an interesting mix of bioactive mixtures like omega‐3 PUFA (polyunsaturated fatty acids), protein hydrolysates enzyme, polypeptides enzyme, peptides, essential amino acids, nutrients, and major minerals (Ashraf et al. 2020).

Customarily, fish is considered a reasonable wellspring of protein, serving an overall populace and a wellspring of nutraceutical significance (Paital 2018). A few bioactive parts in fish, such as fatty lipids, proteins, nutrients, major minerals, and further fish by‐products, are viewed as fundamental because of their helpful medicinal probability (Labudzynskyi et al. 2015). Fish have a wide range of bioactive chemicals with various structures. Much consideration has been paid to peptides with natural exercises and food diversity, which could advantageously affect people. Cell reinforcement and anti‐microbial peptides confined from fish wellspring might be utilized as practical fixings in food production to advance consumer well‐being and further develop the timeframe of realistic usability of food items (Najafian and Babji 2012).

A bioactive compound of fish gelatin is the genre of bio‐polymers procured from the breakdown of fish collagen, and it may have bountiful amino acids for nutritious purposes and dietary items. Regular qualities of fish gelatin imply that fish gelatin items are moderately innocuous to the body compared to medical treatments and medications. Subsequently, dietary utilization of fish gelatin might have superb advantages for individuals with persistent infections like hypertension, osteoporosis, and diabetes. In addition, specialists have tracked down various courses for managing fish gelatin items, like outer use or infusion (Lv LinChen et al. 2019).

Because of their phenomenal biocompatibility, simple biodegradability, and low antigenicity, collagen and gelatin are generally utilized in food, drug, and corrective areas. Fish collagen and gelatin are becoming prominent due to their mammalian counterparts' well‐being and religious worries. The collagen structure has been concentrated on utilizing different present‐day advancements, and understanding the primary information ought to be cautiously made. Collagen shape varies depending on the source and season, which can alter its applications and extraction conditions (Liu et al. 2015). Numerous research has studied the bioactivities and natural consequences of collagen, gelatin, and their hydrolysis peptides. Collagen and collagen‐determined items might apply various possible natural consequences for cells in the extracellular grid by fitting food‐determined peptides after ingestion and their recognized nutritional significance as a protein source. This could explain why they are used in nutritional supplements and pharmaceutical treatments (Wang et al. 2024). Moreover, many new applications have been found for collagen and gelatin. Subsequently, this survey covers the ongoing comprehension of collagen, gelatin, and gelatin hydrolysates' construction, bioactivities, and natural impacts and their most recent applications (Liu et al. 2015; Nurilmala et al. 2022).

Gelatin is the water‐soluble protein created from the breakdown of collagen. Gelatin proteins are hetero‐polymers worked from the amino acids (AA) combined by peptide linkages; amide linkages are framed in the buildup response of AA. The arrangement of those AA comprises the essential construction of the proteins. The characteristics of the proteins rely on a succession of the AA and on working how protein chains are collapsed in space, for example, α‐helix, β‐sheets/unordered arbitrary curl structures that are alluded to as auxiliary structures. Eight most proteins do not take on uniform conformities, and full portrayals of their favored three‐dimensional courses of action are characterized as tertiary designs (Etxabide et al. 2016).

Fish oil rich in lengthy chain omega‐3 PUFA unsaturated fats (n‐3 PUFA) has been perceived for some time for its dietary significance. The prevalent dietary wellsprings of n‐3 PUFA are endless fish oil supplements. In numerous nations, typical fish admission is far beneath the suggested least admission of two fish servings 7 days, for example, around 250 mg each day of n‐3 PUFA (Sanders 2000). Cases of fish oil supplements are constantly added to everyday energy consumption, which is not attractive for some individuals. So, an elective method for guaranteeing an ideal n‐3 PUFA admission is required. Advancement of food sources with n‐3 PUFA increases dietary admission of these unsaturated fats to diminish the danger of different infections (Kolanowski and Laufenberg 2006).

Fortification or improvement generally alludes to adding supplements to food from which they were missing or present in unimportant sums. Fortification with the fish oil will furnish medical advantages shoppers expect with additional huge physiological advantages. However, lipid oxidation restricts the use of these oils in handled food varieties and as dietary enhancements in braced food. The chemical properties of fish oil utilized, the sort of food braced, and the food's supplement profile will likewise impact the physiological capacity of n‐3 PUFA. The numerous acknowledgment rules present a significant test in developing enhanced food items, which incorporate the expansion of a functioning fixing, item newness, tactile qualities, appearance, capacity conditions, simplicity of planning, and wellbeing norms (Jeyakumari et al. 2016).

This review aims to investigate the health advantages of the bioactive chemicals present in Seafood. In order to demonstrate the significance of seafood consumption in enhancing human health, averting chronic illnesses, and bolstering general well‐being, this analysis will examine the nutritional makeup of other seafood species. This overview also aims to thoroughly grasp the health effects of the bioactive substances found in seafood. In addition to highlighting possible directions for further investigation, it aims to compile the most recent study findings on the benefits of bioactive chemicals obtained from seafood for better health outcomes, such as cardiovascular health, anti‐inflammatory qualities, and cognitive function.

## 2 Seafood Distribution

Fish possess the most noteworthy situation in marine creature utilization and are crucial for the global economy. In 2012, fish provided roughly 17% of the global protein necessities, with herrings, salmon fish, cod, fumble fish, and mullet being the most widely recognized types of fish utilized for food (Turunen et al. 2013). The biggest industrially canned fishery item worldwide is tuna fish (e.g., *Thunnus obesus* ). As per the Food and Agriculture Organization of the United Nations (2010), the total catch of the business fish breed extended from 162,981 metric tons in the year 1950 to more than 4.1 million metric tons in the year 2007 (Zampelas et al. 2005).

**FIGURE 1:**
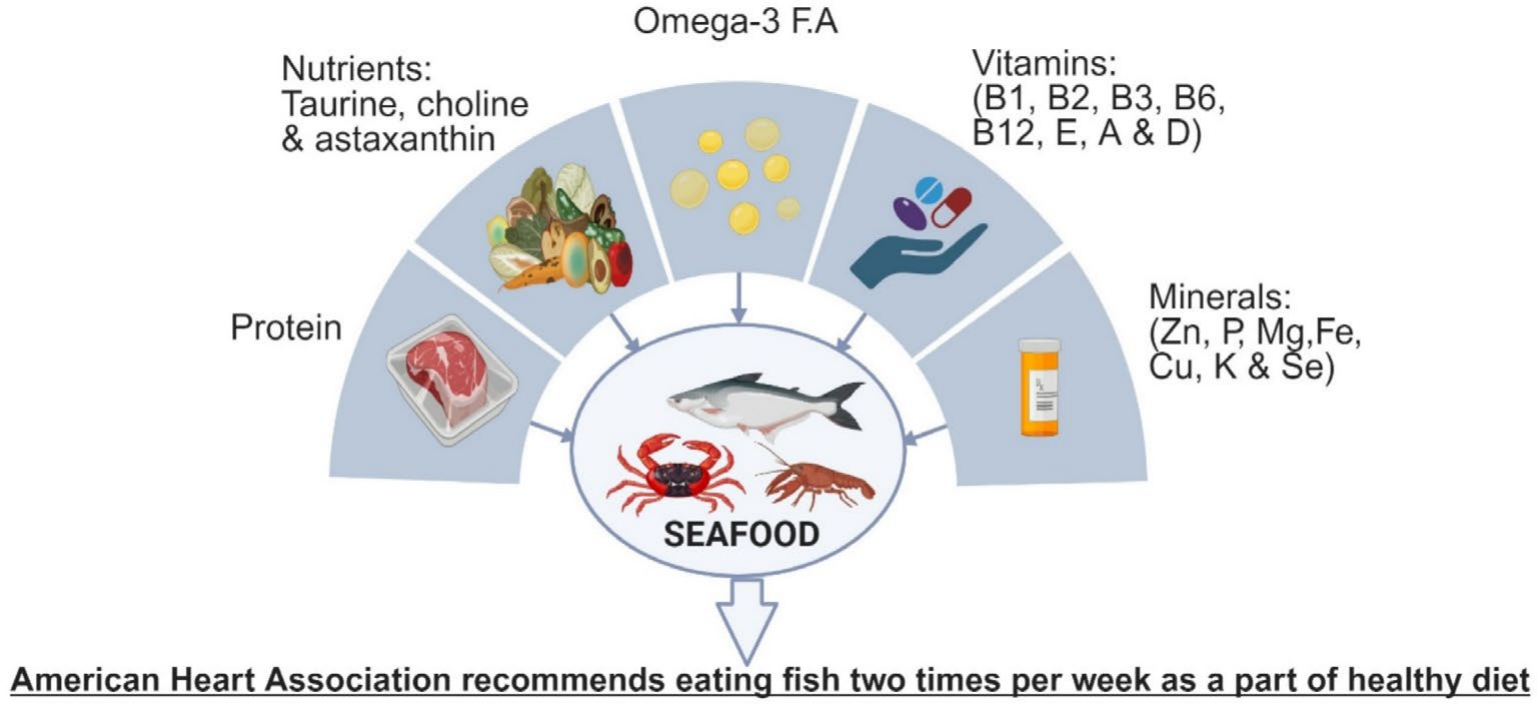
Seafood nutritional composition (Siscovick et al. 2017).

Wholesome advantages of fish utilization are because of the existence of proteins, unsaturated fats, minerals (e.g., Ca, Fe, Sn, and Zn), and nutrients, specifically Vitamins A, B3, B6, B12, E, and D (Figure 1). Analysis has additionally revealed that peptides obtained from matured fish subsequent to enzymatic therapy might be helpful in therapeutics for the treatment of numerous normal intense as well as persistent illnesses such as viral contaminations, hypertension, malignant growth, and Alzheimer's disease. Fish collagen might also be utilized in bone therapy as an option in contrast to mammalian collagen, which is best known as immunogenic (Tsoupras et al. 2022).

## 3 Seafood‐Derived Components

Ocean environments give different scopes of bioactive particles with broad uses as nutraceuticals in food and supplement businesses. Those bioactive atoms can be proteins, peptides, polysaccharides, unsaturated fats, polyphenols, probiotics, chemicals, nutrients, and minerals. Excess areas of this article examine the physical and compound properties of these various atoms and how they contribute to bioactivity in nutraceutical applications (Table 1) (Gil and Gil 2015).

**TABLE 1:** Seafood derived compounds.

Compound	Origin	Effect	References
Omega‐3 fatty acids	Shellfish and fatty fish (sardines, mackerel, and salmon)	It has anti‐inflammatory properties on blood vessels, decreases blood pressure, and lowers triglycerides	Olgunoglu (2017)
Astaxanthin	Salmon, shrimp, and krill	Antioxidant, anti‐inflammatory, beneficial to the skin and eyes	Barros et al. (2014)
Chitin and Chitosan	Shellfish (shrimp, crab)	Reduces fat absorption by binding to fat molecules and improves the healing of wounds	Hayes et al. (2008)
Fucans	Brown seaweed	It has antiviral properties, lowers inflammation, and prevents the formation of clots	García‐Ríos et al. (2012)
Fucoxanthin	Brown seaweed	It lowers blood glucose levels, boosts fat oxidation, and improves lipid metabolism	Méresse et al. (2020)
Taurine	Shellfish, fish	Stabilizes cell membranes, promotes the production of bile salts, and controls blood pressure	Hano et al. (2017)
Collagen	Scales, fish skin	Enhance skin hydration, promote cartilage growth, and quicken the healing of wounds	Alves et al. (2017)
EPA and DHA	Fatty seafood (sardines, tuna)	Control inflammation, promote brain growth, and guard against heart disease	Renuka et al. (2016)
Squalene	Olive oil with shark liver oil	It neutralizes toxic chemicals, strengthens the immune system, and is an antioxidant	Revill et al. (2022)
Phospholipids	Krill oil and fish roe	It sustains cell membranes' integrity, promotes neurotransmission, and raises cognitive performance	Ahmmed et al. (2022)
Vitamin D	Fatty fish (mackerel, salmon)	It improves calcium absorption, boosts immunity, and controls mood and mental health	Yerlikaya et al. (2022)
Iodine	Shellfish and seaweed	Vital for the synthesis of thyroid hormone and controls metabolism	Smyth (2021)
Peptides	Fish and shellfish from the sea	Free radical scavenging, ACE (angiotensin‐converting enzyme) inhibition, and immune system stimulation	Harnedy and FitzGerald (2012)
Sterols (Marine)	Seaweed and shellfish	Reduce the gut's absorption of cholesterol and improve cardiovascular protection	Bakar et al. (2019)
Polyphenols	Algae and seaweed	Reduce the growth of cancer cells, control blood sugar levels, and neutralize free radicals	Jimenez‐Lopez et al. (2021)

### 3.1 Bio‐Lipopeptides

Bioactive bio‐peptides are the protein sections going in sort from 2 to 20 amino corrosive buildups, which might be produced from rear proteins through assimilation or handling. Two elements can impact the kind of bioactive bio‐peptides delivered: (a) The essential succession of the protein catalyst and (b) the explicitness of the enzymes used to produce such peptides. Moreover, bioactive peptides can be created from the proteins utilizing hydrolysis (corrosive/antacid), cooking/maturation. These peptides have a scope of bioactivities, including antimicrobial, immune‐modulatory, antithrombotic, and antihypertensive movement. They are viewed as exceptionally huge mixtures (Toldrá et al. 2018).

An investigation is executed to comprehend the peptide construction, piece, and succession. Bioactive bio‐peptides have different administrative capacities on unambiguous cell‐focused plans. Numerous analysts have zeroed in on creating drug mixtures from ocean‐determined peptides, especially for the ACE restraint and antihypertensive capacity. Ocean proteins from fish, mollusks, and shellfish are among the most extreme wellsprings of bioactive iotas reported that fish protein enzyme hydrolysates have a couple of novel peptides that can bind to cell surface receptors and update Ca ingestion (Li et al. 2023). Supportive usage of these peptides is the treatment of osteoporosis and Paget's disease. Collagen is a significant piece of cow‐like meat used in organizations, such as in excellent care items, pharmaceutics, food, and biomedicine. Meat protein collagen is a fabulous wellspring of bioactive peptides that are antihypertensive and antithrombotic, and inhibitors of brush line intensify like the dipeptidyl peptidase‐IV (Gormley 2006).

### 3.2 Polysaccharides

Marine polysaccharides have many commercial food, beverage, and supplement applications. Fucans/fucanoids, carrageenans, hydro‐colloids, and glycosaminoglycans are marine polysaccharides from green algae, scavengers, and other marine species. These mixtures show antiviral, anticoagulant, antiproliferative, antithrombotic, and mitigating properties. Carrageenans and alginates are direct biopolymers in red and earthy‐colored green growth, respectively, and are distinguished as the most abundant polysaccharides (Xiong et al. 2022; Ruocco et al. 2016).

Other than alginate, earthy‐colored green growth contains exceptionally intricate and sulfated lattice polysaccharides called fucoidans. The saccharide synthesis, sulfate fixation, and different attributes of fucoidans acquired from different ocean breeds shift in places of sulfated gatherings, atomic weights, linkage mode, and grouping of saccharide deposits. Fucoidans' natural qualities are further developed by underlying sulfate gatherings, permitting them to be utilized as nutraceuticals in the dairy business. Secondary metabolites obtained from the sea have numerous health benefits for humans, allowing them to be used as nutraceuticals (Gil and Gil 2015).

### 3.3 Phenolic Compounds

Phenolic chemicals in ocean algae are mostly familiar as an oxidative stress adaptation mechanism. In most marine brown algae (Phaeophyceae), phlorotannins are the most abundant polyphenols, though flavonoids make up the greater part of the general polyphenolic content in dark green algal growth. The dark brown algal phlorotannin profile chiefly comprises phloroglucinol, eckol, and dieckol. The antioxidant movement has additionally been accounted for by phlorotannin, empowering these phenolic mixtures to be utilized as dynamic fixings in nutraceuticals (Arnold and Targett 2000).

Carotenoids, as polyphenols, are delivered by specific marine microbes and green growth and have cell reinforcement qualities, expanding their utility as nutraceuticals. Carotenoids are normal shades with a 40‐carbon structure that are lipid‐dissolvable. Prebiotics are nonedible, specifically matured synthetics that advance the development and action of solid stomach microbes, which help the host's well‐being. Prebiotics are oligosaccharides, such as chitosan oligosaccharides, whereas other algal polysaccharides are likewise known to have prebiotic movement. Bifidogenic gains have likewise been accounted for from exopolysaccharides created by ocean lactic corrosive microscopic organisms (Olaniran et al. 2023).

Carotenoids can neutralize the free radicals in three steps which include electron transfer (oxidation, reduction: CAR + ROO → CAR^+^ + ROO^−^), hydrogen abstraction (CAR + ROO → CAR + ROOH), and addition (CAR + ROO → ROOCAR) (Milani et al. 2017).

Additionally, the cyanobacterial biomass of Limnospira platensis (formerly Spirulina platensis) can invigorate bacteria (*Lactobacillus*) and bacteria (*Bifidobacterium*) species, advancing their prebiotic impact. Dark Red and Dark blue‐green growth, amphibian plants, microalgae, and kelp produce photosynthetic colors. These shades give nutraceutical specialists regular food shading and calming, anticarcinogenic, and cancer‐preventing agent compounds (Simopoulos 2008).

### 3.4 Enzymes, Vitamins and Minerals

Enzymes can change particles into significant biotechnological devices in food and nutraceutical enterprises. Proteins can impact waste, capacity, handling, and security as food fixings. Lipase enzyme, chitinolytic compounds, enzyme polyphenol oxidase (Catecholase enzyme, tyrosinase enzyme, cresolase enzyme, polyphenolasev, catechol oxidase enzyme, phendase enzyme), transglutamase enzyme, and dark red‐colored algal proteins associated with the starch corruption process (e.g., – 1, 4‐glucanase) are catalysts obtained from marine sources (Gadallah et al. 2023).

They have magnificent compound, physical and synergist properties contrasted with their earthly partners. They can additionally be disabled at modest temperatures and reveal high synergist action when the temperature is low. Furthermore, catalysts are utilized as foodadditives. Because of their salt tolerance and specificity, they are employed in food processing with various characteristics and soaring movement at gentle pH biomolecules. For example, chemicals segregated from extremophiles can be profoundly helpful in food products because of their exceptional exercise under strange circumstances (Olaniran et al. 2023).

Extremophiles have long been brought to the table for incredible potential as biotechnology assets. Vitamins and minerals play various roles in the body, including providing transport within cells and acting as cofactors in metabolic cycles. Kelp is high in iron, iodine, manganese, and zinc, among other vitamins and minerals. Iodine could be obtained naturally from some seaweed species (Alfio et al. 2021).

## 4 Utilization and Demand for Seafood

Asia represented 66% of human utilization; 36.8 million tons were consumed outside China, and 33.7 million tons were consumed in China. The typical fish utilization per capita for North America, Central America and the Caribbean, South America, Oceania, and Europe was 24.2, 9.6, 8.5, 20.9, 24.6, and 20.7 kg, respectively (FAO 2010). Fish utilization changed by more than 100 overlays between various world regions and between the inland and waterfront districts of nations (Wadhwa and Bakshi 2013).

Throughout recent years, the food security of fish has been improved because of mechanical improvements in handling, appropriation, transportation, and capacity. These upgrades are acknowledged as cost‐effective and have improved security and standards. Besides, improving the enormous scope, significant distance refrigerated transport and quicker bale revived worldwide exchange. It brought about utilizing a more extensive assortment of species and new fish. Consumers demand top quality, convenience, reliability, and well‐being in developed countries. Shoppers in these countries likewise search for food with well‐being‐advancing characteristics (Hosomi et al. 2012).

### 4.1 Seafood Consumption

Hundreds of millions of people in developing countries rely on aquaculture and fisheries. Overfishing, including taking fish beyond sustainable levels, reduces fish stocks and employment worldwide (Baset et al. 2020). Fisheries play a crucial part in Pakistan's economy and are a fundamental wellspring of nourishment, pay, and employment (Jarwar 2008). The healthy benefit of fish consumption is exceptionally elevated, with a protein compound of 15%–20%, low cholesterol content, and numerous supportive dietary enhancements (Table 2) (Izquierdo et al. 2001).

**TABLE 2:** Major bioactive compounds, consumption, and distribution of seafood (FAO 2020).

Seafood types	Major bioactive compounds	Consumption (%)	Distribution	References
Tilapia	Omega‐3, Omega‐6, Phosphorus, Selenium, and Protein	12	Egypt, China, Indonesia, and the USA	El‐Sayed and Fitzsimmons (2023)
Salmon	Vitamin D, B12, astaxanthin, EPA, DHA, and omega‐3	10	Canada, Japan, the USA, and Norway	Onozaka et al. (2023)
Tuna	Protein, Vitamin B12, Omega‐3 (EPA, DHA), and Selenium	8	USA, Indonesia, Japan, and Spain	Paquotte and Lem (2008)
Cod	Vitamin D, protein, iodine, selenium, and omega‐3	7	Portugal, Iceland, Iceland, Norway	Trondsen (2012)
Pollock	Phosphorus, Protein, Omega‐3, and Vitamin B12	6	USA, China, Japan, and Russia	Pramod et al. (2014)
Sardines	Calcium, vitamin D, protein, antioxidants, and omega‐3	5	Portugal, Spain, Italy, and Morocco	Basurco et al. (2014)
Herring	Omega‐3 (EPA, DHA), protein, and vitamin D	5	Russia, Poland, Germany, and the Netherlands	Toner (2015)
Shrimp/Prawns	Protein, B12, vitamin D, zinc, and astaxanthin	9	Thailand, USA, India, China	Shinn et al. (2018)
Catfish	Phosphorus, Vitamin D, Protein, and Omega‐3	4	Nigeria, China, Vietnam, and the USA	Lawrence et al. (2022)
Mackerel	Protein, Coenzyme Q10, Vitamin D, and Omega‐3 (EPA, DHA)	4	Spain, Korea, Japan, and the UK	Paquotte and Lem (2008)
Anchovies	Calcium, selenium, protein, vitamin D, and omega‐3	3	Greece, Italy, Spain, and Turkey	Dinçer (2018)
Trout	Protein, Vitamin B12, Vitamin D, and Omega‐3	3	Japan, the UK, Norway, and the USA	Karnai (2018)
Shellfish (Mussels, Oysters, Clams)	Protein, Iron, Zinc, Omega‐3, and Vitamin B12	9	Japan, France, the USA, and Spain	Paquotte and Lem (2008)

A survey was conducted by Baset et al. (2020) in District Dir Lower in Khyber Pakhtunkhwa Province of Pakistan. The survey was conducted through an adequately structured questionnaire from March 13 to June 2, 2019, to determine the consumption of fish (i.e., Mahasheer, trout, catfish, Common Carp, Rohu, etc.) according to educational levels, gender, per capita, based on species liking, size, time duration, and the order of preference given by the people. The current survey results showed that fish consumption had no relation to the educational level because illiterate and educated males consumed 75.51% and 58.53% of fish, respectively.

Similarly, the percentage for illiterate females was 66.67%, and for educated females was 55.17%. The per‐head consumption for the lower class was 0.49 kg/year, followed by the middle class at 0.46 kg, then the upper class at 0.37 kg/year. The percentage of persons who consumed fish monthly was 46.25%, whereas only 8% annually. The people who avoided fish for its spines and the high cost were 50.75% and 31.25%, respectively. It was observed that people know that fish‐eating can increase eyesight, help with high blood pressure, and reduce cardiac disease by 46.75%, 13.25%, and 25%, respectively (Baset 2020; Osman et al. 2001).

*Channa striata* is ordinarily eaten as a food fish. In Malaysian countries, freshwater fish utilization provides a fundamental wellspring of protein, comprising more than 71% of complete protein, and it is also recognized as a wellspring of omega‐3 unsaturated fats. *Channa striata* is included unmistakably in nearby eating routines among the Malays, the Orang Asli of Peninsular Malaysia (Haemamalar et al. 2010; Kodoh et al. 2009).

## 5 Medicinal Effects of Seafood

*Channa striata* are committing air‐breathing freshwater fish that possess a wide range of water bodies from little trenches to rice fields and streams across tropical and subtropical Asian nations from country Pak and India to Southeast Asia and Southern China (Hossain et al. 2008). The therapeutic impacts of *Channa striata* are ascribed to two significant parts: the AAs and the unsaturated fats. AA's earliest reported concentrate on the corrosive profile of Channa striatus was directed in 19s on fileted *Channa striata* separate. The review tracked down the concentrate rich in glycine, a trivial amino corrosive. Later examinations have affirmed this outcome (Dahlan‐Daud et al. 2010).

Other unimportant AAs that have all the earmarks of overflow in *Channa striata* remove incorporate amino acids, such as glutamic acid, arginine, and aspartic acid (Shafri and Abdul Manan 2012). These AAs significantly affect the feeling of agony and mending wounds. A concentrate on the protein content of the bodily fluid of *Channa striata* reveals that variety exists in various filtration types. Rough concentrate seems to contain the most noteworthy measure of protein (0.589 mg/mL) trailed by the fluid (0.291 mg/mL) and acidic concentrates (0.291 mg/mL) (Wei et al. 2010).

The review, nonetheless, did not dissect the nitty gritty amino acid synthesis of *Channa striata* bodily fluid—unsaturated fats. The capacity of *Channa striata* to deliver unsaturated fats, for example, Eicosapentaenoic acid (EPA) and Docosahexaenoic acid (DHA) (Jaya‐Ram et al. 2011) *Channa striata* is likewise exceptionally esteemed for its therapeutic properties. Of the many kinds of fishes in Malaysia, just the Malaysian channidae (counting Channaa microplates, *Channa striata* and *Channa gachua* ), the mudskipper, *Periophthalmus* spp., and the Anguillidae, *Monopterus albus* are known to be utilized in conventional Malay medication. Other Southeast Asian groups, such as the Thais, Vietnamese, Cambodians, and Chinese, additionally use *Channa striata* to treat infections (Shafri and Abdul Manan 2012) (Table 3).

**TABLE 3:** Different types of seafood their effects and nutrients.

Type	Effects	Nutrients	References
Salmon	It lowers inflammation and heart disease risk and supports brain function	Omega‐3 fatty acids (DHA and EPA), protein, and vitamin D	Lund (2013)
Tuna	It stimulates muscle recovery, lowers cholesterol, and improves cardiovascular health	Protein, selenium, vitamin B12, and omega‐3	Senevirathne and Kim (2012)
Sardines	It strengthens the immune system, maintains heart health, and enhances bone health	Calcium, vitamin D, vitamin B12, and omega‐3	Santos et al. (2023)
Mackerel	It lowers blood pressure, boosts brain and cognitive function, and has anti‐inflammatory properties	Vitamin B6, selenium, vitamin D, and omega‐3	Wang et al. (2014)
Shrimp/Prawns	It enhances skin elasticity, promotes cognitive health, and helps with weight management	Protein, iodine, selenium, and astaxanthin	Menon and Lele (2015)
Oysters	It boosts libido, strengthens bones and cardiovascular health, and strengthens the immune system	Iron, zinc, omega‐3, and vitamin D	Negara et al. (2022)
Clams	It increases vitality, promotes cardiovascular health, and enhances mental performance	Iron, B12, protein, and omega‐3 fatty acids	Bassey et al. (2014)
Crab	It supports healthy skin, strengthens bones, and enhances brain function	Copper, zinc, protein, and omega‐3	Maulvault et al. (2013)
Lobster	It increases energy, improves neurological function, and synthesizes red blood cells	Copper, phosphorus, omega‐3, and vitamin B12	Nguyen et al. (2017)
Cod	It lowers inflammation, encourages lean muscle mass, and supports heart health	Omega‐3, iodine, selenium, and lean protein	Senevirathne and Kim (2012)
Tilapia	It strengthens the immune system, aids tissue healing, and supports bone health	Potassium, selenium, phosphorus, and protein	Van Doan et al. (2019)
Herring	It reduces blood pressure, promotes mental well‐being, and enhances metabolism	Vitamin D, protein, selenium, and omega‐3	Lund (2013)
Mussels	It strengthens the immune system, increases circulation, and benefits joints	Vitamin B12, iron, selenium, and omega‐3	Grienke et al. (2014)
Seaweed	It possesses anticancer qualities, enhances intestinal health, and optimizes thyroid function	Fucoxanthin, dietary fiber, antioxidants, and iodine	Kumar and Sharma (2021)
Squid	It enhances energy generation, strengthens the immune system, and supports the health of the muscles	Copper, protein, phosphorus, and vitamin B12	Kim and Pallela (2012)
Krill	It enhances joint health, lowers inflammation, and enhances cardiovascular performance	Phospholipids, astaxanthin, and omega‐3	Ulven and Holven (2015)

## 6 Potential Risks Associated With Seafood Consumption

Fish are commonly contaminated by substances like methyl mercury, polychlorinated biphenyls (PCBs), dioxins, pesticides, and plastic waste. Other pollutants include arsenic, cadmium, lead, selenium, polycyclic aromatic hydrocarbons (PAHs), and chlorinated hydrocarbon pesticides. These contaminants originate from both natural and human‐made processes. Naturally, metals such as mercury and arsenic are released from the earth's crust, while human activities like the production of industrial chemicals, pesticide usage, agricultural runoff, and improper waste management introduce pollutants into the environment. Additionally, PCBs, formerly used extensively in industrial operations, contribute to the contamination (Demelash Abera and Alefe Adimas 2024). RAAS and sustainable sourcing.

The Risk Assessment for Sourcing Seafood (RASS) was launched in September 2014. By September 2016, the platform featured 360 fishery profiles and recorded approximately 8000 annual visits. It is utilized by various businesses, including processors, food services, retailers, and restaurants, to guide procurement decisions and inform customers about the environmental impacts of specific fisheries. Developed by the UK Sea Fish Industry Authority, RASS is a web‐based tool designed to educate UK seafood businesses and retailers about four key environmental risks associated with sourcing wild‐capture seafood: fish stock status, management effectiveness, bycatch, and habitat impact. These risks are evaluated on a 5‐point scale (1 indicating very low risk and 5 indicating very high risk) based on criteria outlined in this framework. Unlike other advisory tools or “fish lists,” RASS does not explicitly recommend “buy” or “avoid” but instead helps seafood buyers align their choices with corporate social responsibility (CSR) goals (Caveen et al. 2017).

## 7 Bioactive Compounds in Seafood

### 7.1 Protein

Proteins are composite polymers comprised of a blend of 20 distinct AAs coded by the quality (DNA) code alongside a few other AAs. Proteins are critical to science and food frameworks, working as chemicals and moving proteins and antibodies. They are likewise required to be healthy. The body quickly digests and absorbs fish proteins (Hamed et al. 2015). Hydrolysates due to protein hydrolysis have been getting escalated examination. Those peptides were detached from different fish species (Ibañez et al. 2011).

Digestive enterocytes consume them into the circulatory system, where they apply useful natural movement on body capacities and conditions. Peptides open up new and good roads for fostering a different scope of biotechnological items with improved bio‐active properties. These have been outlined with a huge scope of organic movement, including antimicrobial, cell reinforcement, and resistance to hypertensive properties (Kris‐Etherton et al. 2004).

Fish protein enzyme hydrolysates (FPH) are hydrolyzed proteins obtained from fish species such as fish, mackerel, and others. They have shown cell reinforcement action. Two fundamental AAs found in enormous amounts in fish proteins are lysine and methionine. Angiotensin I changes over protein (ACE) inhibitory peptides from fish sources, which were first connected with sardine meat over 20 years ago. Pro‐inhibitory peptides have since been found in different fish species, including shellfish, bonito, salmon, and sardine. Various reports have reported rough fish protein enzyme hydrolysates containing ACE inhibitory peptides obtained from sanitized catfish protein hydrolysis. The solvent protein contained most ACE inhibitory peptides delivered (Ryan et al. 2011).

Fish muscles overflow with amino acids, principally glutamic corrosive, proline, taurine, glycine, alanine, and arginine, among the water‐soluble parts. Fish is a decent wellspring of taurine, a conditionally essential amino corrosive engaged with specific aspects of mammalian development. The molecule contains a sulfonic corrosive gathering, as opposed to the carboxylic corrosive moiety, that is not integrated into the proteins and is perhaps the most copious free AAs in many tissues, including skeletal and cardiovascular muscle and the brain. Taurine fish is available in cod, mackerel, cultivated and wild salmon, tuna fish, beam, shark, whiting, and a few other species. There is a potential use of taurine to reduce blood pressure, improve cardiovascular performance, and lower blood cholesterol levels (Kadam and Prabhasankar 2010).

In affirmation, European specialists have demonstrated that taurine benefits CV well‐being and fish is a decent source. Crafted by Gormley has revealed a steady disparity in the taurine substance of four breeds in the recorded request as “plaice (126), cod (93), mackerel (69), and cultivated salmon (53 mg/100 g).” It suggests that white fish have more taurine than sleek Fish. Free AAs typically collaborate with the free revolutionaries. The most productive ones can undoubtedly offer hydrogen particles, including the AA's nucleophilic sulfur‐containing side chains—cysteine and methionine or fragrant side chains (Tryptophan, Tyrosine, and Phenylalanine). This infers particular mixtures liable for the bioactivity of fish hydrolysates (peptides/amino acids) are AA cysteine, AA methionine, AA lysine, AA taurine, AA tryptophan, AA tyrosine, and AA phenylalanine. Glutamic corrosive, proline, glycine, alanine, and arginine are also used. Accordingly, endless fish separates are hostile to hypertensive peptides (ACE inhibitors), which can bring down circulatory strain and frustrate plaque or cholesterol stores on the internal surfaces of the corridors, deterring the bloodstream (Kundam et al. 2018).

Tarine helps protect the central nervous system by lowering the endoplasmic reticulum stress and acts as a antagonist for the neurotransmitter receptors of GABA, glycine and NDMA. Through the process of cell regulation the taurine safeguards the cardiovascular system by regulating the Calcium transport, ROS generation, and protein phosphorylation. It also mitigates the diabetes mellitus symptoms. Taurine acts as an anti‐inflammatory substance by forming taurochloramine in neutrophils and prostaglandins mitigating effects of rheumatoid arthritis and osteoarthritis (Schaffer and Kim 2018).

### 7.2 Lipid and Fatty Acids

Substance cosmetics of fish oil vary from oils of different origins. It contains two sorts of unsaturated fat: Docosahexaenoic (DHA) and Eicosapentaenoic (EPA). These are polyunsaturated, unsaturated fats named omega‐3 unsaturated fats and are predominantly found in fish species with higher unsaturated fat contents (Alvarez and Rodríguez 2000). Despite immersed fats, the PUFA in fish oil is readily processed for energy production and is reported to have various bioactivities. Scientific researchers believe that EPA and DHA are two key protective compounds of fish oil that prevent chronic disease. Analysis results show that the ingestion of fish raises the levels of EPA and DHA in blood, consistently lowering the incidence of coronary heart disease through various mechanisms (Harris et al. 2013).

Bringing down the movement of coronary illness has led to the declaration that these unsaturated fats in fish oil are associated with avoiding sicknesses in people. Fish oil has likewise been found to have a defensive effect Against heart sicknesses by diminishing serum fatty substance levels, further improving heart work, bringing down circulatory strain, and diminishing irritation (Kim and Mendis 2006).

Fish contains Polyunsaturated fatty acids and significant measures of MUFA, which are considered valuable if not oxidized (Larsen et al. 2011). Thus, the main particular bioactive fish oil mixtures are Eicosapentaenoic (EPA) and Docosahexaenoic (DHA). This is additionally affirmed by Scientist Gormley, who has revealed that fish ingestion can neutralize aggravation in cardiovascular well‐being. This is associated with a diminishing risk of cardiovascular illnesses through omega‐3 polyunsaturated fatty acids (PUFAs), made chiefly from eicosapentaenoic and docosahexaenoic corrosive. That reduces the degree of platelet collection in the blood, diminishing blood and the propensity to form blood clusters (Kundam et al. 2018).

It has been investigated by Zheng et al. (2013) that “the recommended minimum daily intake of EPA/DHA varies from 250 to 1250 mg.” Also, different specialists attested that the lipid part of fish is comprised principally of PUFAs, which are fundamental to human well‐being. People are unequipped to blend PUFAs and have longer than 18 carbon particles; hence, they get them from food. The amalgamation of long‐affixed PUFAs happens in the green growth eaten by fish, making fish the superb wellspring of long‐tied PUFAs for people (Lordan et al. 2011). The long‐chain omega‐3 unsaturated fats safeguard against cardiovascular sickness (CVD). They are primarily present in the brown‐colored tissue of sleek fish, which contains most oil. Essential business wellsprings of omega‐3 PUFAs are fatty fishes such as sardine, fish herring, salmon, and mackerel (Sijtsma and De Swaaf 2004).

### 7.3 Saturated Fatty Acids (SFA)

SFA has been connected with a higher fatty substance (TG), complete cholesterol (TC), and lessened‐thickness lipoprotein (LDL) cholesterol levels in the blood. Huge groupings of SFA in cell layers and lipoproteins make them less functional. Such lipoprotein particles make stable associations with cell lipoprotein receptors, bringing about issues with the human body's cholesterol transport framework and resulting in dyslipoproteinemias, which contribute to atherosclerosis (atherogenic dyslipoproteinemia). In this way, SFA utilization is normally connected with the seriousness of atherosclerotic sores of the conduits and the advancement of CVD and other persistent sicknesses, whereas SFA ought to be diminished in food sources and food items, including supplements, to keep away from such metabolic illnesses (Stanley et al. 2012).

The unsaturated fat piece of fish is fundamentally portrayed by a low satisfaction of SFA, which has been suggested as an advantage of consuming fish or fish oil over other creature‐inferred protein and oil sources. C15:0 (myristic corrosive), C17:0 (palmitic corrosive), and C16:0 (stearic corrosive) are the most widely recognized SFA found in fish, representing 9‐half of absolute unsaturated fats. The unsaturated fat synthesis, made up of 34 ocean water fish breeds, was likewise answered to go from 30.11% to 46.89% SFA, with palmitic and stearic corrosive being the significant corrosive SFA. For most of the 34 marine water breeds in examinations, items in these soaked unsaturated fats were as per the following: myristic corrosive (C14:0, 0.73%–8.09%), palmitic corrosive (C16:0 15.97%–31.04%), and stearic corrosive (C18:0 2.79%–11.21%) (Chen et al. 2022).

### 7.4 Monounsaturated Fatty Acids (MUFA)

Over the last many years, mono‐unsaturated fats have been perceived as possibly helpful for decreasing cardiovascular disease risk. Most of the exploration on the medical advantages of MUFA has zeroed in on the effects of oleic corrosive and palmitoleic corrosive. It is indistinct whether MUFA with chain lengths longer than 18 carbons can assist with persistent sicknesses (Eroldoğan et al. 2013). Early exploration in Greenlandic Inuit Eskimo uncovered that a high admission of food varieties wealthy in both PUFA and MUFA long‐chain n‐3 PUFA and MUFA, like fish, have cardio‐protective characteristics, suggesting a possible connection between lengthy chain n‐3 PUFA and MUFA consumption as well as the diminished hazard of cardiovascular diseases (CVD), that is as yet dubious because the Inuit's diminished sickness hazard could be credited to hereditary fluctuations in their eating routine (Özogul and Özogul 2007).

Oleic corrosive safeguards against cardiovascular insulin obstruction by working on endothelial brokenness because it favors provocative signs and diminishes multiplication and apoptosis in vascular smooth muscle cells that might contribute to an improved atherosclerotic process and plaque dependability. Additionally, it was found that oleic corrosive is connected to incendiary pointers and cardiovascular breakdown. Simultaneously, oleic corrosive has also been found to have areas of strength for thrombotic intensity by hindering platelet conglomeration. Regardless, more studies on the effects of MUFAs, such as oleic corrosive, on cardiovascular disease risk factors and medical endpoints are needed to determine if MUFAs can play a role in primary and secondary CVD prevention (Aarón et al. 2022).

### 7.5 Polyunsaturated Fatty Acids (PUFA) and n‐6/n‐3 PUFA Ratio

PUFAs are unsaturated fats with at least two twofold securities. The most notable PUFA classes are n‐3 and n‐6 PUFA; the trademark of unsaturated fats of this class are n‐3 ALA and n‐6 LA, which are essential PUFAs. The existence of a twofold bond at the third or sixth carbon from the methyl end of the unsaturated fat chain indicates n‐3 or n‐6 unsaturated fat classification, respectively. Both ALA and LA are essential unsaturated fats, meaning that the human body cannot produce them. Other long‐chain bioactive PUFAs, such as the n‐6 PUFA arachidonic corrosive (ARA) and n‐3 PUFAs EPA and DHA, can be created (Strobel et al. 2012).

Then again, people cannot integrate ALA. Moreover, we cannot change it entirely to EPA or DHA, requiring dietary wellsprings of this long‐chain n‐3 PUFA, such as greasy fish. The advantages of endless fish oils have been certified for their high n‐3 PUFA materials and their anti‐thrombotic and anti‐inflammatory properties for some time. n‐3 PUFA applies to both anti‐atherogenic and hostile thrombotic impacts. It has been proposed that fish n‐3 PUFA act fundamentally as antecedents to different anti‐inflammatory synthetic substances, lessening the creation and tissue mix of the n‐6 PUFA ARA and its provocative eicosanoid subordinates. Moreover, specific cyclooxygenases (Coxs), as well as lipoxygenases (LOXs), produce less favorable to fiery prostaglandins and leukotrienes from EPA than those produced by ARA (Alfio et al. 2021).

#### 7.5.1 Taurine

The proportion of n‐6/n‐3 PUFA in an eating routine can be significant because n‐3 PUFA causes and expands calming eicosanoids that function as adversarial inhibitors to the provocative eicosanoids acquired from n‐6 PUFA. Compared to our progenitors' eating regimen, the proportion of n‐6/n‐3 PUFA in the Westernized diet has risen decisively. Irritation, fatness, and other connected diseases are on the ascent because of these severe changes. The proportion of the Western eating regimen went from 15 to 16.7 in 2002. It is by now around 20:1 (Calder 2001).

Compared with the eating routine, wherein people developed and where their hereditary patterns were laid out, Western diets have a low admission of n‐3 PUFA and unnecessary amounts of n‐6 PUFA. Subsequently, the lower the n‐6/n‐3 PUFA proportion in food sources like fish or one's whole eating regimen, the better the well‐being result against persistent aggravation and apoplexy. Subsequently, fish's high n‐3 PUFA content and calming properties play a fundamental role in the dietary well‐being benefits of endless fish oil (Duarte 2024).

Hence, polyunsaturated fatty acids of marine origin, especially the long‐chain n‐3 PUFA such as EPA and DHA, have been checked for their impacts on a few irritation‐related illnesses, including CVD, malignant growth, Alzheimer's disease, diabetes, and a few central nervous system problems, whereas PUFA, especially DHA, was beneficial to the brain and optical systems. Assisting with preventing coronary disease, the recent examination has likewise hypothesized that using EPA and DHA may influence the functions of the immune and reproductive systems (Kamiloglu et al. 2021).

Aside from free amino acid, taurine (2‐aminoethanesulfonic corrosive) is available in all tissues and plentiful in the heart, blood, retina, and brain. Taurine‐manufactured activity in humans is more vulnerable than in guinea pigs and rodents, and dietary reliance on taurine is high. Thus, taurine is an unnecessary, however, restrictively essential, amino acid substance in the human body. Taurine plays numerous fundamental roles in several critical biological processes, such as calcium regulation, bile acid formation, antioxidation, membrane stabilization, and immunity. Humans consume taurine primarily through fish, which contains higher amounts of taurine than meat. Generally, taurine is especially abundant in several marine invertebrates: clam tissue has multiple/100 g of taurine contents. However, the taurine contents in terrestrial plants are low or absent (Sahar and Rahman 2018).

Taurine has advantageous antihypertensive, antihypercholesterolemic, and mitigating impacts on lifestyle‐related infections. Moreover, human intervention studies have uncovered that the administration of taurine and n‐3 PUFAs has hypolipidemic and antiatherogenic impacts compared to n‐3 PUFA supplementation alone. In non‐DM fat human subjects, 3 g/day taurine supplementation greatly diminished serum TG, the atherogenic index, and BW compared to a fake treatment bunch (Hosomi et al. 2012).

### 7.6 Carotenoids

Salmon, shellfish, and shrimp squander might be a fundamental wellspring of carotenoids (e.g., astaxanthin). On that side, the admission of carotenoids from food or supplementation ought to be painstakingly viewed as the absence of explicit biosynthetic pathways in people. Then again, Carotenoids are captivating bioactive synthetics that can assist with decreasing the unfavorable impacts of oxidative pressure. Specifically, a good connection between increased dietary admission, tissue groupings of carotenoids, and less hazard of ongoing infections was present (Tan et al. 2024).

In addition, cell reinforcement movement of astaxanthin regulates natural capacities connected with lipid peroxidation, gainfully affecting persistent illnesses like cardiovascular diseases, macular degeneration, and cancer. In the specific instance of astaxanthin, its oral organization of 1.1 mg/kg/day for 14 days significantly decreased hepatic metastasis in rodents, showing that prevention of stress‐induced lipid peroxidation plays a fundamental role in supporting the immune response. With increasing separate focuses, astaxanthin and its esters showed high antioxidant action. Moreover, astaxanthin repressed the expansion of human laryngeal cancer cells (Hep 2 cells). Curiously, to further improve astaxanthin's stability from the shrimp shells ( *Litopenaeus vannamei* ), the encapsulation in alginate‐chitosan globules was attempted to permit its genuine use as a functional ingredient (Atef and Ojagh 2017).

### 7.7 Fiber

Largely, muscle‐based fish contain insignificant starches and fiber. Palatable kelp contains a great deal of dietary fiber (26%–75% dry weight), and water‐dissolvable fiber is roughly 52–85. In light of their pigmentation, kelp is ordered into three fundamental gatherings. Earthy‐colored kelp is transcendently dark brown due to fucoxanthin and has essential polysaccharides, for example, fucans, cellulose, alginates, and laminarins (Moreno et al. 2002).

Dark green kelp is green because chlorophyll and ulvan are critical polysaccharide parts. Dark Red Ocean growth has phycoerythrin and phycocyanin as their chief shades; they additionally have agars and carrageenans as the essential polysaccharides. In creature studies, polysaccharides extracted from the different edible ocean growth have been found to diminish total cholesterol, low‐density lipoprotein (LDL) cholesterol, and TG in plasma (Rioux et al. 2017).

### 7.8 Phytosterols

The design of phytosterols is like cholesterol, with just minor contrasts in an overall place of ethyl and methyl gatherings. Phytosterols are normal fixings in plants, and the chief structures are β‐sitosterols, stigmasterol, and campesterol. Types of phytosterols in marine spineless creatures incorporate free sterols, stanols, and sterol esters (Kanazawa 2001).

Phytosterols are frequently used to foster quality food, including low‐ and fat‐free yogurt, milk, juices, spreads, cereals, and bread. Medical preliminaries have reliably shown that admission of 2.1–3 g/day of phytosterols is related to a vast lowering (somewhere in the range of 4.2% and 15%) of blood LDL cholesterol (Comunian and Favaro‐Trindade 2016). Phytosterols have caused the hypocholesterolemic impacts related to an admission of specific consumable microalgae, and microalgae have been sent off as modern makers of phytosterols. The lipid‐lowering component of phytosterols is remembered to happen when phytosterols contend with the retention of cholesterol by binding to micelles in the digestive tract (Rozner and Garti 2006).

Thus, their presence in the digestive system hinders cholesterol dependability in micelles, diminishing cholesterol retention. One more part of phytosterols is that they upgrade the enterocyte ATP‐restricting tape (ABC) G5 and ABCG8 proteins, which discharge cholesterol into the gastrointestinal lumen and articulation (Corrêa et al. 2017). Phytosterols have likewise been accounted for as being answerable for other biochemical properties, including calming, cell reinforcement, and anticancer impacts. Scarcely any examinations have analyzed the connection between high‐portion phytosterols and decreased fat–solvent nutrients, cell reinforcements, and carotenoids. Extra exploration was expected about phytosterols' food handling and accessible enhancements in the human body (Simopoulos 2008).

## 8 Most Useful Discards From Several Seafood Taxa

In light of the great substance of proteins, polyunsaturated fatty acids (PUFA), and different supplements with different medical advantages, such as carotenoids, minerals, nutrients, squalene, and glycosaminoglycans, fish squanders are utilized as a natural substance for fish feast, and compost, or as a part of water and chicken takes care of. Accordingly, notwithstanding the low worth of fishery side effects, there is developing interest in their possible use as practical parts, nutraceuticals, and drugs in different applications. On one hand, this technique has a critical monetary advantage, as it creates extra income from a material that, in certain circumstances, has a removal amount. On the other hand, valorizing fish can decrease contamination, such as bycatch and other results (Table 4) (Kris‐Etherton et al. 2019; Zeller et al. 2018).

**TABLE 4:** Useful discards from different seafood species and their utilization.

Specie	Discard part	Utilization	References
*Mercenaria mercenaria*	Shells	Utilized in crafts, building supplies, soil conditioners, and calcium supplements	Creswell and McNevin (2008)
*Octopus vulgaris*	Tentacles and heads	Utilized to make pet food, fish bait, food processing, and marine collagen	Shahidi et al. (2019)
*Sardina pilchardus*	Bones, viscera, and heads	Production of fishmeal, fish oil, fertilizers, and biodiesel	Caruso et al. (2020)
*Engraulis encrasicolus*	Bones, viscera, and heads	Fish oil, fishmeal, fertilizers, and animal feed	Saleh et al. (2022)
*Placopecten magellanicus*	Gonads and shells	Gonads are utilized as decor, biofilters, shells in calcium products, and specialty meals	Gurr et al. (2024)
*Crassostrea gigas*	Shells	Utilized in the manufacture of jewelry, aquaculture substrates, lime, and water filtration	Elegbede et al. (2023)
*Xiphias gladius*	Head, cartilage, and skin	Adhesives, fishmeal, collagen extraction, and cartilage for supplements	Kumar et al. (2012)
*Scomber scombrus*	Bones, viscera, and heads	Fishmeal, fertilizer, animal feed, and fish oil	Shirai and Ramirez‐Ramirez (2010)
*Salmo salar*	Offal, heads, bones, and skin	Seafood oil, fishmeal, skin‐derived collagen, bone gelatin, and biofuel	Siddiqui et al. (2023)
*Gadus morhua*	Skulls, skins, and liver	Fishmeal, liver for cod liver oil, and skins for collagen and gelatin	Shirai and Ramirez‐Ramirez (2010)
*Thunnus albacares*	Fins, skin, and bones	Shark fin soup (fins), fishmeal, collagen, fish gelatin, and biofuel	Cutajar et al. (2022)
*Homarus americanus*	Heads and shells	Animal feed, nutritional supplements, and bioplastics all use chitin extraction	Mathew et al. (2021)
*Portunus pelagicus*	Claws, exoskeletons, and shells	Manufacturing of chitin and chitosan, which are utilized in medicine and water treatment	Wahab et al. (2023)
*Loligo vulgaris*	Mantle, beak, and ink	Beak and mantle for gelatin, collagen, and animal feed; ink for natural dyes	Gajendra et al. (2020)
*Penaeus monodon*	Heads, tails, and shells	Shrimp oil, animal feed, flavor enhancers, and chitin extraction	Abuzar et al. (2023)

Since fishery and hydroponics squanders are high in excellent supplements, the marine bioprocess industry has a colossal chance to change over and utilize an enormous part of these significant items. For example, fish represents a rich wellspring of proteins differing in functional and organic properties; high fixations can be found in fish heads, spines, and tails, among different spots (Djoussé et al. 2012). These proteins and different mixtures tracked down in fish, especially shellfish, can be effectively removed using novel biotechnological advances, such as the chitosan obtained from the shrimp *Pleoticus muelleri* exoskeletons. These progressions incorporate (1) macromolecule biotransformation through proteins or microorganisms, (2) subcritical and supercritical extractions for target item seclusion, (3) ultra‐filtration, (4) microwave, and (5) ultrasound‐assisted recuperation strategies, and film partition. Accordingly, modest and energy‐efficient enzymatic methodologies in light of proteases, glycoside hydrolases, lipases, and trans‐glutaminases are arising in food handling (Alfio et al. 2021).

Executing, de‐shelling, cleaning, destroying, balance and scale evacuation, fileting, washing, and other fish handling squanders. This waste can represent up to 50% of all fish consumed. This content can be wasted as substantial disposals of refuse or results. The level of waste materials delivered changes depending upon the handled organic entity; for instance, finfish can create up to half of the waste material, including digestion tracts, heads, skeletal edges, skin, scales, and viscera. During fish canning tasks, the cycle creates a higher level of strong squanders (around 70 for every cent) (Cooper et al. 2016).

Prehandling cycles of fish from fisheries and hydroponics create an assortment of squanders, contingent upon the unrefined substance and the ideal eventual outcomes in different markets. Specifically, astaxanthin is monetarily utilized as a cell reinforcement and a feed element for further improving tissue coloration in hydroponics (i.e., pink shade) of cultivated salmonids, which is well known among clients (Fiori et al. 2012).

Marine green growth squanders could be taken advantage of, notwithstanding fish and shellfish. In actuality, palatable ocean growth is high in minerals and nutrients and is a phenomenal wellspring of iodine and one of a handful of plant wellsprings of vitamin B12. Numerous Asian recipes incorporate (Chlorophyta), (Rhodophyta), *Undaria*, *Laminaria*, *Himanthalia*, and *Saccharina* (Phaeophyceae), assisting with working on the nature of various food items. Since macroalgae squanders are deficient in satisfying global needs, different algal breeds are seriously grown, especially in coordinated hydroponics (Alfio et al. 2021).

## 9 Health Benefits of Seafood Consumption

### 9.1 Cardio‐Protective

**FIGURE 2:**
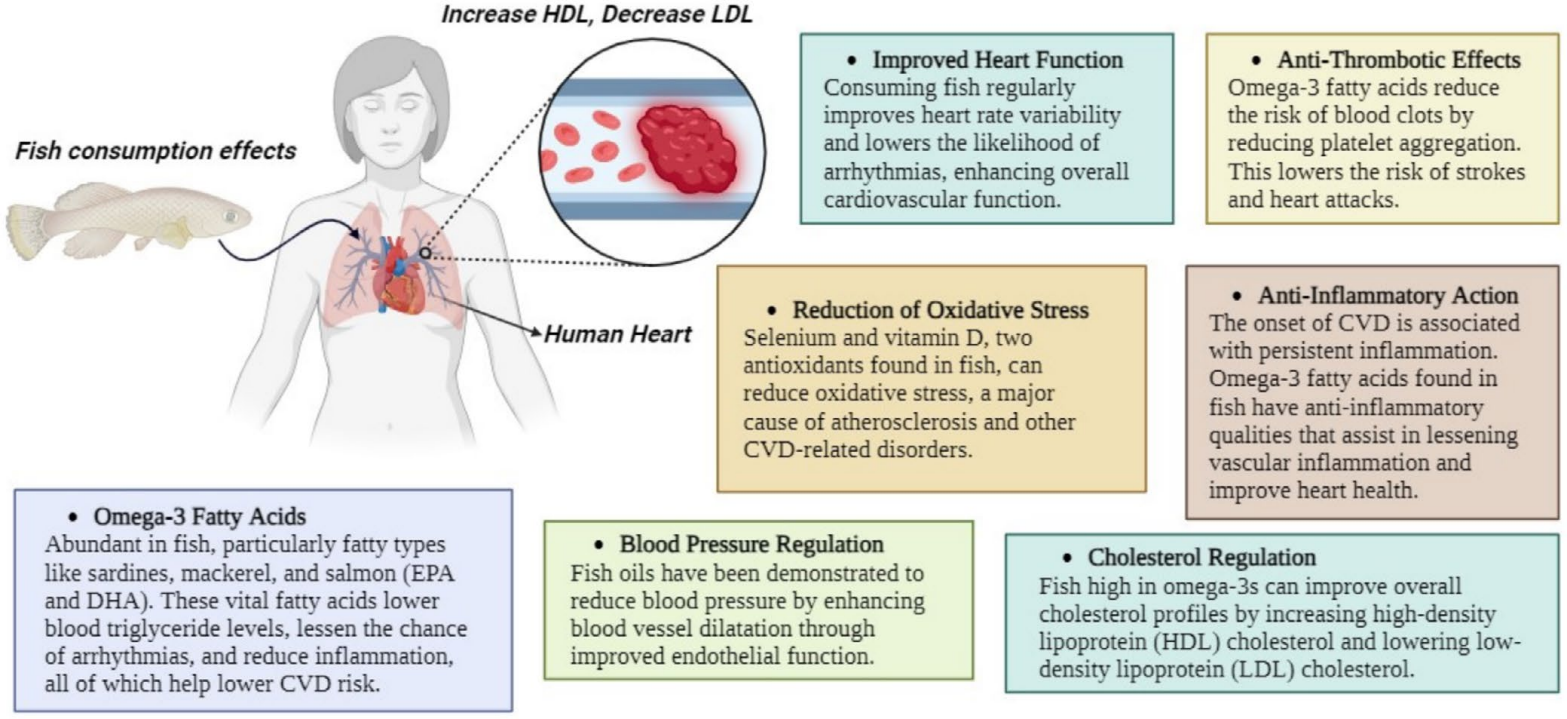
Fish consumption effects on heart health.

In the United States and numerous different countries, cardiovascular sickness (CVD) is a primary source of dismalness and passing (Li et al. 2025). CVD counteraction is a general well‐being point that envelops a few systems, one of which might incorporate remembering fish for one's eating routine. Expanding fish utilization is suggested for the admission of omega‐3 (n‐3) unsaturated fats and gives benefits for the gamble decrease of cardiovascular sickness (CVD) (Figure 2). Most Americans are not accomplishing admission levels that consent to current recommendations. The reason for this study is to give an expansive image of the issues influencing admission deficiency. Thus, we depict the connection between fish admission and CVD risk decrease. The other wholesome commitments of fish to the eating regimen diminished CVD risk with fish consumption, especially for the diminished chance of death from heart occasions. Fish species giving elevated degrees of EPA and DHA might be generally defensive against CVD (Raatz et al. 2013). The AHA 2006 Diet and Lifestyle Recommendations educate utilization regarding no less than two servings of fish each week, ideally greasy fish high in DHA and EPA.65 The rules likewise prescribe an everyday fish consumption comparable to 1 g/day of EPA and DHA for optional counteraction of CAD. Fish oil supplements containing EPA and DHA are recommended compared to greasy fish utilization for auxiliary prevention (Weitz et al. 2010).

A randomized experiment assessed the effect of suggesting fish consumption on CHD in male patients with the prevalent illness. This UK experiment offered nutrition guidance under an open‐label design. A factorial design was used to randomly assign men (*n* = 2033) who had suffered a myocardial infarction within the last 1.5 months to receive advice on eating two servings of fatty fish per week, reducing total fat to < 30% calories, and increasing the ratio of polyunsaturated to saturated fat to 1.0, or increasing cereal fiber to 18 g/day. About 20% of randomized participants who got fish advice instead consumed 1.5 g/day of fish oil. After 2 years, neither the dietary fat nor the cereal fiber groups showed any discernible differences compared to the control group. Relative risk (RR), 0.73; 95% confidence interval (CI), 0.56–0.95, and CHD mortality (RR, 0.68; 95% CI, 0.51–0.91) were significantly lower in those who were randomized to receive fish guidance. Participants who were still living and were initially randomized to get fish guidance reported eating more fish but significantly less, according to a follow‐up conducted 15 years after the trials (Burr et al. 1989, 2003).

Concerns have been expressed that consuming large amounts of n‐6 PUFAs may reduce the health advantages of LC n‐3 PUFAs received from Seafood. Intake of n‐3 and n‐6 PUFAs does not interact much, according to several prospective studies that have looked into this matter. In the Health Professionals Follow‐up Study, men with low (RR, 0.52; 95% CI, 0.34–0.79) or high (RR, 0.60; 95% CI, 0.39–0.93) n‐6 PUFA intake were found to have a 40%–50% decreased risk of sudden cardiac death if they consumed ≥ 250 mg/day of n‐3 PUFA (P for interaction = 0.13) (Virtanen et al. 2008).

Virtanen et al. (2012) conducted a study in Finland that looked at 91 cases of sudden cardiac death involving 1857 men. They found that men with low hair mercury had a 23% lower risk of sudden cardiac death (RR, 0.77; 95% CI, 0.64–0.93), but men with high hair mercury did not show any significant effects (RR, 1.02; 95% CI, 0.95–1.09; P for interaction = 0.01). According to the Cardiovascular Health Study, older persons who ate tuna or other broiled and baked fish had a 40% decreased chance of having an ischemic stroke; on the other hand, those who ate fried fish or fish sandwiches had a higher risk (Mozaffarian et al. 2003).

It was challenging to identify if the type of fish caused the variation in lean fish low in n‐3 PUFAs or the preparation technique of frying in partially hydrogenated oils. Two sizable cohort studies of Chinese men and women were merged to investigate the relationship between seafood consumption and stroke mortality. According to that investigation, people who ate more LC n‐3 PUFAs and saltwater fish were at a decreased risk of dying from ischemic stroke (Takata et al. 2013).

If people substituted Seafood for processed meat for two meals a week, their risk of coronary heart disease (CHD) would be significantly reduced. However, if they substituted healthy vegetarian meals for the two seafood meals a week, their estimated risk reduction might be substantially lower or nonexistent. Recent research of two sizable US cohorts found that substituting 3% of processed meat's total protein calories with 3% of Seafood's total protein calories was linked to a 31% decreased risk of cardiovascular mortality (Song et al. 2016). Substitution models are a more accurate way to quantify the hazards and benefits of consuming Seafood compared to particular alternatives, and they should be considered in future individual studies and systematic reviews.

### 9.2 Antidiabetic

**FIGURE 3:**
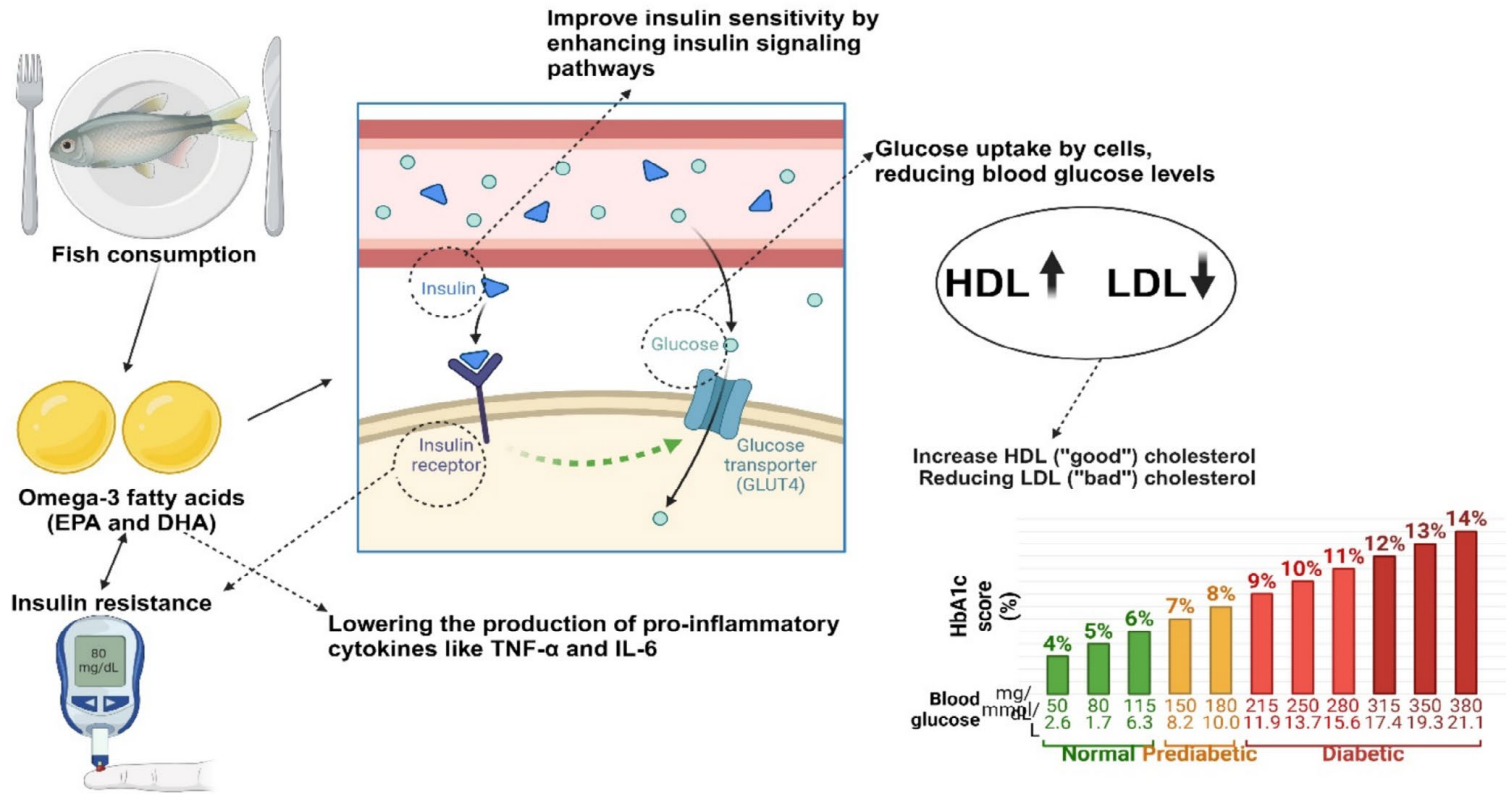
Fish mechanism of action in managing DM.

Fat‐less fish protein counts calories utilized in creature studies have lowered insulin obstruction, showing that such weight control plans would oversee human glucose (Figure 3). As an affirmation, cod‐protein consideration in a more‐fat and sucrose diet safeguarded movement of weight related to insulin opposition and glucose resilience as exhibited because of insulin responsiveness tries that glucose amount expected to prompt hyperglycemia was essentially higher for cod‐took care of rodents than casein‐took care of rodents. Cod protein further improved insulin‐subordinate glucose take‐up, highlighting improved insulin responsiveness in contrast to casein. The expanded insulin awareness was connected to the degree of fat stores and weight gain that were brought down. A similar example was seen with cod protein taken care of by humans (Tastesen et al. 2014). An inverse effect of consumption of oily fish and diabetes mellitus was seen, and it was seen that the risk of T2DM was reduced by 38.6% (Zhang et al. 2024).

Nkondjock and Receveur (2003) conducted an ecological study examining 41 nations across five continents, each with unique sociodemographic traits and hygienic circumstances. We gathered information from websites on food balance sheets, the prevalence of obesity and diabetes, and other related topics. An investigation of their interaction followed correlations between the variables under study. There was a significant connection (*ρ* = 0.81, *p* < 0.0001) between the prevalence of type 2 diabetes in the 20–44 and 45–64‐year age groups after adjusting for total energy intake. Compared to the 20–44 age group, type 2 diabetes was approximately five times more common in the 45–64 age group. Diabetes, obesity, and total fish and seafood consumption interacted. The prevalence of type 2 diabetes rose considerably with obesity in nations with low fish and seafood consumption (0.8% ± 0.3% vs. 2.5% ± 1.8%; *p* = 0.002 and 3.3% ± 2.6% vs. 11.0% ± 3.9%; *p* < 0.0001 for the 20–44‐ and 45–64–year age groups, respectively). There was evidence of considerably lower type 2 diabetes with high fish and seafood consumption in countries where obesity was more common (2.5% ± 1.8% vs. 0.9% ± 0.7%; *p* = 0.007 and 11.0% ± 3.9% vs. 6.2% ± 4.1%; *p* = 0.041 for the 20–44‐ and 45–64–year age groups, respectively).

### 9.3 Anti‐Cancer

Prostate disease frequency and mortality fluctuate up to 60‐overlay universally. Even though distinctions in symptomatic force might make sense as part of the variety, the significant variety in mortality across nations may likewise propose the job of lifestyle and dietary variables in its goal (Dewailly et al. 2003). This thought is upheld by the critical expansion in prostate malignant growth rate and mortality among workers from okay to high‐take a chance with countries. The job of fish admission and prostate disease has been concentrated on in a few settings. Populations with maximum usage of fish, like populations in Japan and Alaskan Eskimos, have lower paces of prostate malignant growth than populations with Western weight control plans, in which fish admission is mostly lower. Fish are rich in long‐chain marine omega‐3 polyunsaturated fatty acids, which might reduce prostate disease hazard and movement by diminishing fiery processes (Szymanski et al. 2010). Fish oil supplementation had cancer mortality of (*p* < 0.001) with 1.6% increase in deaths in nonusers and glucosamine showed (*p* < 0.01) with 3.4% increase in mortality in nonusers (Lam et al. 2024).

### 9.4 Anti‐Obesity Effect

**FIGURE 4:**
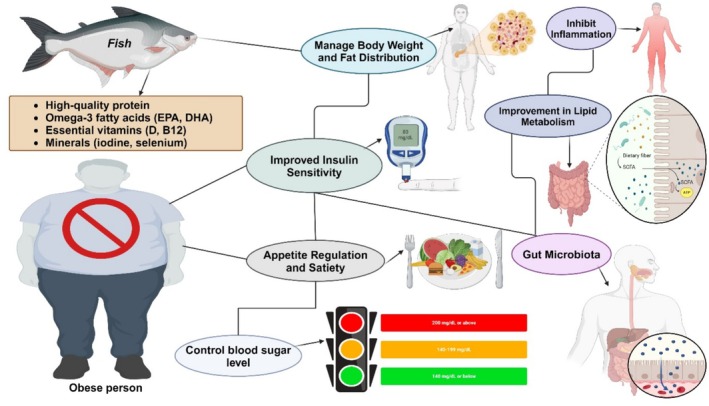
Fish aiding in obesity.

Obesity has turned into a worldwide scourge because of hereditary weakness, expanding access to high‐energy food choices, and diminishing the requirement for active work in current life. Obesity should not be excused as a simply stylish issue influencing a few individuals. Specifically, stoutness is related to DM, coronary illness, certain kinds of malignant growth, and rest breathing problems (Liu et al. 2024). Corpulence is characterized by a weight file (weight separated by the level's square) of 30 kg m^−2^ or more prominent. However, this does not consider the dismalness and mortality related to additional unassuming levels of overweight or the hindering impact of intrastomach fatness. Be that as it may, a pandemic undermines worldwide prosperity (Figure 4) (Kopelman 2000).

Fish has been portrayed as an equipped cell reinforcement source since its structure offers a lower measure of soaked fat than numerous other food things. It is rich in cancer prevention agent substances, particularly in a few amino acids, for example, taurine. Notwithstanding, the creation of fish frequently incorporates delegate PUFA sums, for example, omega‐3 unsaturated fats, whose substance structure makes them reasonable for peroxidation, even though it very well might be guaranteed that this favorable to oxidant impact could be enhanced by cell reinforcement parts occurring in the fish flesh (Parra et al. 2007).

Numerous studies have detailed the positive effects of marine n‐3 polyunsaturated fatty acids on obesity‐related illnesses. Because of this, fatty fish like mackerel, salmon, and herring have long been considered healthful because of their high marine n‐3 PUFA concentration. Since eating a lot of meat is linked to gaining weight, eating fish and shellfish is related to losing weight (Smith et al. 2015).

### 9.5 Brain Health

**FIGURE 5:**
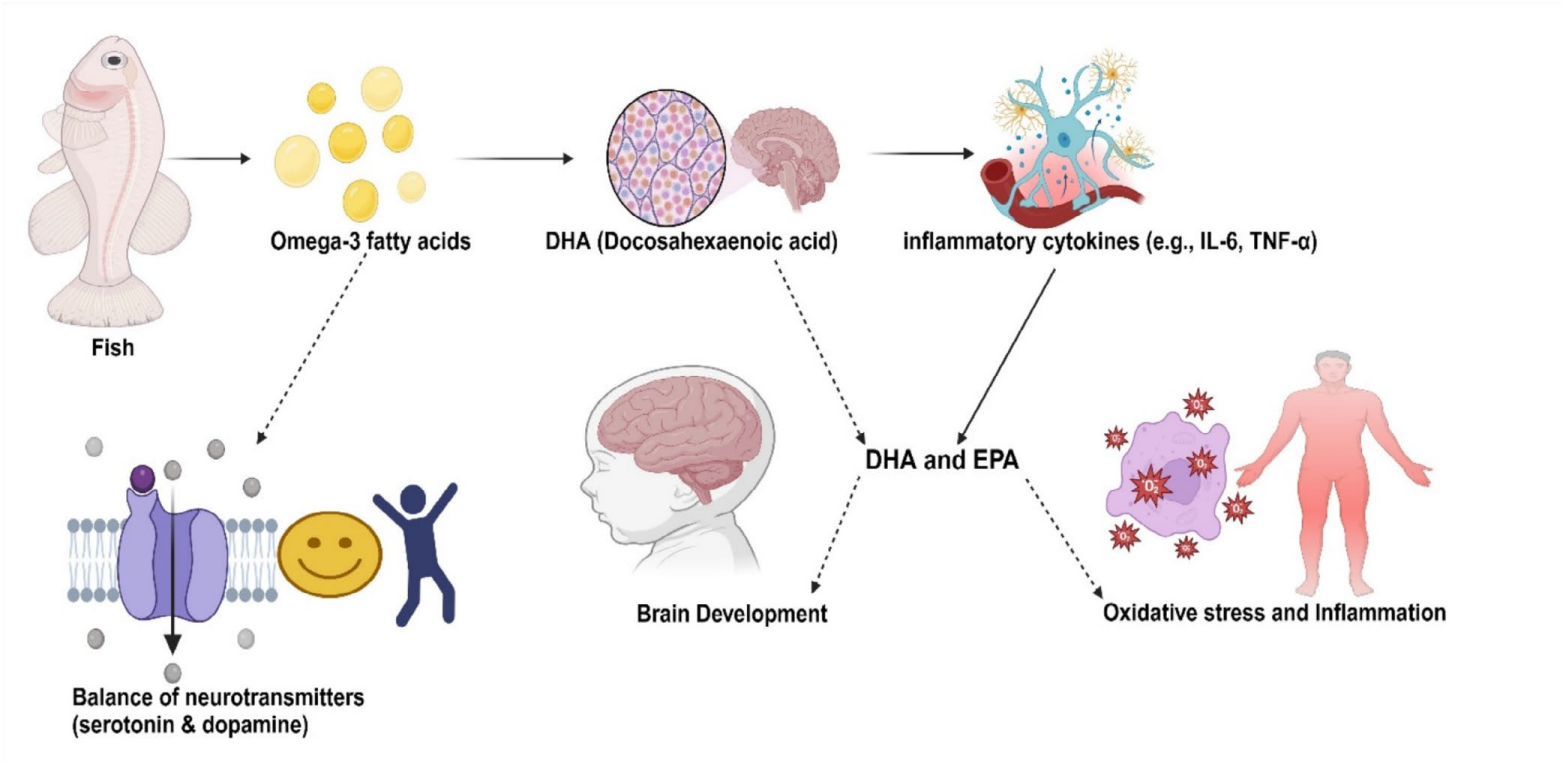
Fish consumption effects on brain health.

Discouragement bringing down, first‐stage improvement, and mental errands conservation in the advanced period are essential regions where fish ingestion is favorable to the human cerebrum (Figure 5). These important advantages are accomplished through omega‐3 unsaturated fats (PUFAs) found in fish oil and the various characteristic cell reinforcement parts that generally forestall oxidative harm of the PU fatty acids in the fish (Mozaffarian and Rimm 2006).

Standard dietary fish admission is demonstrated to bring down marks of wretchedness in patients. The good connection between fish consumption and fetal turn of events and the start of life is moored on the tremendous amounts of DHA mixed into the mind and eye retina. The firm, direct pressing of DHA is incredible because of the underlying edge of the cis‐twofold bonds, so huge amounts of this unsaturated fat result in upgraded smoothness of the cerebrum and retina cell films (Dórea 2008).

Hence, the DHA‐caused liquid cell structure is proposed to work on the ideal execution of the organs by expanding the skill of flagging go b/w—a couple of like particles, eicosanoids, and sugars move vital all throughout the cells. Improved execution of the mind and retina was seen when premature children were cared for with DHA‐enhanced eats less. In contrast, the ones without DHA lacked legitimate mental and retina capacities. Likewise, moms with fish oil‐enhanced slims down during pregnancy had children who grew up showing unrivaled mental and critical thinking capacities, including hand and eye development. For safeguarding mental undertakings during more established ages, fish oil has been displayed to relieve scholarly disintegration (Assisi et al. 2006).

Omega‐3 unsaturated fats in fish oil are associated with mental health and are fundamental for vision and the conceptive framework. This can be credited to DHA since it is a part of the mind nerve neurotransmitter in the eye retina, testicles, and male sperms. In this manner, the Omega‐3 unsaturated fats of fish oil achieve a fundamental capacity in the turn of the event and execution of the mind, regenerative framework, and so forth (Duhan et al. 2020).

### 9.6 Anti‐Oxidative Activity

Cell reinforcement alludes to intensities that can prevent oxidations by out‐of‐cost revolutionaries in the human body when present in low fixations. Hence, cancer prevention agents can safeguard our body against oxidative pressure and the progression of disorders. Fish protein hydrolysates from fish breeds, for example, fish, mackerel, fish yellowfin sole, and Alaska Pollock have shown cell reinforcement abilities. The hydrolysates or peptides extracted from fish are from the muscles, viscera, skin, bones, and scales (Yin et al. 2025; He et al. 2013). Fish hydrolysates and peptides have antioxidant qualities that are good for health because they shield the body from reactive oxygen species (ROS) and reactive nitrogen species (RNS), which can damage proteins, lipids, and DNA and cause degenerative diseases like dementia, diabetes, cancer, and obesity. The evidence that fish hydrolysates and peptides scavenge free radicals produced by ROS and stop ROS‐induced oxidation of cell membranes is consistent with these findings. Also, peptide size and amino corrosive profile determine the viability of fish protein hydrolysates or peptides (Chalamaiah et al. 2012).

As of late, normal cancer preventives acquired from food sources are becoming famous because of endeavors to separate from the potential risks of manufactured cell reinforcements. A couple of instances of fish showing potential cell reinforcement proof are as follows: Fish protein hydrolysates/peptides from mackerel were seen to exhibit cancer preventives. The cell reinforcement capacity of fish protein hydrolysate was displayed by its capacity to forestall hydroxyl extremist prompted DNA harm. Similar to a well‐established reality, DNA harm is liable for the commencement of a few terminal diseases, such as coronary illness, diabetes, malignant growth, etc. Thus, standard fish admission would forestall or treat such disorders (Atef and Ojagh 2017; Shaik and Sarbon 2022).

### 9.7 Maternal Care

Marine long‐chain omega‐3 unsaturated fats are undoubtedly connected with pointers of fruitfulness in all kinds of people. Notwithstanding, fish, their essential food source, can likewise be a wellspring of poisons, which could check the regenerative advantages. The positive relationship between fish admission and fertility was more grounded for couples when the two accomplices ate high fish during follow‐up (Hinton and Miller 2013).

Overall, the assessed rates of pregnant couples by 6 and a year among the couples who devoured at least eight fish servings for every cycle were 82% and 91%, contrasted with 63% and 78% among the couples consuming less. It is converted into an adapted to of 1.62 (96% CI, 1.16–2.22); Supplemental and a 14% lower outright distinction in the rate of fruitlessness. The positive relationship between male, female, and couple fish admission and fruitfulness was marginally constricted after the change for SIF; notwithstanding, the FORs for the most elevated female and couple fish customers remain measurably significant (Gaskins et al. 2018).

## 10 Plant‐Based Seafood Alternatives

Plant‐based seafood alternatives are designed to replicate the texture and sensory qualities of traditional seafood. Between 2002 and 2021, 149 such products were introduced, with a 244% increase in launches by 2021 compared to 2002. Nutritional comparisons reveal no significant differences in energy, total fat, saturated fat, carbohydrates, and sugar content between tuna alternatives and conventional tuna, both of which have high fat content due to being canned in oil. Shrimp alternatives have similar energy and fat levels but significantly higher carbohydrates and sugars due to starchy ingredients. Vegan fish fingers contain more energy, fat, and salt than vegetarian counterparts but show no major differences in other nutrients (Boukid et al. 2022).

Vegans, vegetarians, and those who follow Kosher dietary laws can enjoy plant‐based seafood alternatives that mimic the taste, texture, and nutritional benefits of traditional seafood without harming animals or violating religious rules. Replicating seafood's texture involves simulating its nanometric fibrous structure, derived from tissue‐, cellular‐, and molecular‐level protein bonds. Some studies have achieved this by incorporating soy and pea protein isolates or concentrates into surimi gels, partially or fully replacing fish‐based ingredients. Examples of seafood alternatives: (i) Good catch: Tuna chunks, fish burgers, fish cakes, and crab cakes made with a six‐legume blend (peas, chickpeas, lentils, soy, fava beans, and navy beans). (ii) Gardein: Fish filets and crab cakes made with soy, wheat, and potato. (iii) Plant‐based foods: Caviar made with seaweeds (Kazir and Livney 2021).

## 11 Conclusion and Future Perspectives

Over 69% of Earth's surface comprises seas, whose rich biodiversity offers a vast supply of natural and medicinal materials. Numerous bioactive compounds that benefit human health can be found in seafood. They can be used in various industries, including food, cosmetics, and pharmaceuticals. Seafoods are easily acquired for the development of functional foods because they are readily available and provide the potential to both prevent and cure certain diseases. Seafood comes in various forms and is eaten for its nutritional value. The sea is considered the most prominent surviving reserve of natural chemicals that can be utilized as functional additives in food products, and it provides a wealth of resources for discovering new compounds. Numerous seafood‐derived bioactive substances have antibacterial, antioxidant, antidiabetic, anti‐obesity, and neuroprotective qualities, among other health advantages. Repurposing fish waste from aquaculture and fisheries into beneficial nutraceuticals effectively addresses waste management issues and improves human health. As a result, appropriate efforts should be undertaken to produce marine functional meals, as consuming them may lessen the incidence and severity of chronic illnesses.

## Author Contributions

**Tabussam Tufail:** methodology (equal), writing – original draft (equal). **Huma Bader Ul Ain:** methodology (equal), project administration (equal). **Jawad Ashraf:** methodology (equal), supervision (equal). **Sammina Mahmood:** data curation (equal), validation (equal). **Sana Noreen:** formal analysis (equal), visualization (equal). **Ali Ikram:** supervision (equal), validation (equal). **Muhammad Tayyab Arshad:** data curation (equal), writing – review and editing (equal). **Muhammed Adem Abdullahi:** conceptualization (equal), writing – original draft (equal).

## Disclosure

The authors have nothing to report.

## Consent

This study did not involve humans.

## Conflicts of Interest

The authors declare no conflicts of interest.

## Data Availability

The data supporting this study's findings are available from the corresponding author upon reasonable request.
